# Sleep Deprivation Increases Mortality Risk Among Older Adults with Epilepsy

**DOI:** 10.3390/healthcare13090977

**Published:** 2025-04-23

**Authors:** Srikanta Banerjee, Jagdish Khubchandani, Stanley Nkemjika

**Affiliations:** 1 College of Health Sciences, Walden University, Minneapolis, MN 55401, USA; 2 College of Health, Education, and Social Transformation, New Mexico State University, Las Cruces, NM 88003, USA; jagdish@nmsu.edu; 3 Department of Psychiatry and Human Behavior, Thomas Jefferson University, Philadelphia, PA 19107, USA; stanley.nkemjika@waldenu.edu

**Keywords:** epilepsy, seizure, sleep, mortality, risk

## Abstract

**Introduction:** Among U.S. adults, over 3 million report a history of epilepsy, accounting for nearly 1.2% of the population. Sleep deprivation is a well-known risk factor for increased likelihood, intensity, and length of seizures. However, the long-term impact of sleep deprivation on people with epilepsy is not well explored. The purpose of this study was to assess mortality risk among individuals with epilepsy based on sleep duration. **Methods:** Data from the 2008–2018 National Health Interview Survey (NHIS) were linked with mortality data from the National Death Index (NDI) for US adults aged 65 years and older. Survival curves showed the combined effect of sleep deprivation and epilepsy, using the Kaplan–Meier product-limit method to estimate the percent survival of the subject at each point in time. **Results:** For all-cause mortality, the unadjusted hazard ratio (HR) for sleep deprivation to no sleep deprivation among people with epilepsy (PWE) was HR = 1.92. The adjusted HR was elevated, HR = 1.94, among individuals who had epilepsy and sleep deprivation but close to 1.0 among individuals who had a history of sleep deprivation without epilepsy after adjusting for demographic and health variables. **Conclusions:** From a nationally representative sample, this first-of-its-kind study in the U.S. found that sleep deprivation and epilepsy combined have worse outcomes than sleep deprivation alone. Clinicians should screen and manage sleep disorders to improve their long-term prognosis of people with epilepsy.

## 1. Introduction

Epilepsy, affecting more than 3 million people in the United States, is defined by continuous, unpredictable, recurrent seizures, with over one-third being uncontrolled by Antiepileptic Drugs (AEDs) [1,2,3,4,5]. Epilepsy is the third most common neurological disorder in the U.S., closely following cerebrovascular diseases and neurodegenerative disorders among older adults (aged > 65 years). The number of older people who develop epilepsy is set to rise substantially worldwide and in high-income countries, where epilepsy incidence is already highest in this age group [6,7,8,9]. As established by the American Academy of Neurology, seizure freedom is defined by persons with epilepsy (PWE) that have gone without a seizure for one year. With the rise in chronic diseases, the major etiological factors in late-onset epilepsy are cardiovascular disease (CVD) and stroke, comprising the cause in up to half of late-onset seizures and epilepsy cases [7,9]. Neoplasms and traumatic brain injuries are some additional reasons for epilepsy that are more common in older adults [8]. However, outcomes may differ for older adults with epilepsy depending on time since diagnosis [7,8,9].

Sleep is responsible for homeostasis through neural, hormonal, and immune support [6]. One-third of a person’s lifetime is spent sleeping or attempting to sleep, making sleep deprivation a major current and prevalent public health issue [10,11,12,13,14]. The National Sleep Foundation, USA, suggests that 7–9 h of sleep is essential for the maintenance and restoration of metabolic homeostasis [10,12,14,15]. About one-third of the U.S. adult population does not meet these recommendations [10,12,16,17]. Several studies have highlighted how sleep disorders profoundly and often exclusively affect older adults, increasing their risk for numerous other health issues [10,11,12,13,18,19,20]. Larsen et al., in a systematic review, purport that PWE are three times more likely to experience sleep–wake disorders than the general population [21,22]. Also, researchers have found, from electroencephalogram (EEG) studies, that sleep deprivation is associated with increasing interictal epileptiform discharges and neuronal excitability, potentially causing the occurrence of seizures. Furthermore, a significant proportion of seizures and increased epileptiform discharges occur during sleep, necessitating the importance of assessing for potential seizures during sleep and investigating the relationship between sleep and epilepsy [23,24,25]. Researchers also found that a tenth of the physiology of sleep is a multifaceted process influenced by hormonal, neurological, psychological, and vascular factors that, in turn, influence overall health. The existing literature supports the relationship between epilepsy and sleep deprivation [26,27]. Despite the recognition that older PWE face unique physical, social, and economic challenges, there is a need to understand the needs of older PWE. In this study, we assessed whether sleep deprivation independently influenced all-cause mortality among PWE in older adults.

## 2. Methods

The National Health Interview Survey (NHIS) is an annual and nationally representative sample of the U.S. population conducted by the National Center for Health Statistics (NCHS). The main purpose of the NHIS is to collect information about individual, household-level indicators and the health characteristics of the civilian, non-institutionalized population in the US. The National Center for Health Statistics (NCHS) developed public-use versions of the NHIS linked with death certificate records from the National Death Index (NDI). For this study, we used the 2008–2018 (extracted from the 2008, 2010, 2013, 2015, and 2017 NHIS Sample Adult components) public-use linked mortality file containing mortality follow-up data from the date of survey participation through 31 December 2019. Each NDI possible match record is assigned a probabilistic match score. The probabilistic match score is the sum of the weights assigned to each of the identifying data items used in the NDI record match, where the weights reflect the degree of agreement between the information on the submission record and the NDI death record. The NHIS data used here were downloaded through the Integrated Public Use Microdata Series (IPUMS) of the Minnesota Population Center. The IPUMS NHIS data are based on the Center for Disease Control and Prevention/CDC’s original data collected but are recoded to study the characteristics of people within the context of families and co-residents.

### 2.1. All-Cause Mortality

We examined the increased risk of overall mortality, as measured by hazard ratios, by using the International Classification of Diseases 10th revision (ICD-10). Follow-up time for individuals who died during the study period was estimated by the number of months from the month/year of interview to the month/year of death. Since the NHIS-NDI database provides only the quarter of deaths, we assumed that death occurred in the middle of the quarter, February, May, August, or November.

### 2.2. Sleep Deprivation

Sleep deprivation was assessed by asking participants the following question: “On average, how many hours of sleep do you get in a 24 h period?” Researchers have previously used sleep deprivation as a proxy for low sleep duration [27]. Participants estimated habitual sleep duration by describing the number of hours. Sleep duration was categorized into 3 groups, short sleep duration (<6 h), normal sleep duration (6–8 h), and long sleep duration (>8 h), consistent with consensus recommendations provided by the American Academy of Sleep Medicine (AASM) and Sleep Research Society (SRS). Also, according to the National Sleep Foundation, a healthy sleep duration is no less than 7 h for adults. For this study, we considered <7 h of sleep for each study participant as short sleep duration [10,12,18,28].

### 2.3. Persons with Epilepsy PWE

The NHIS epilepsy module that we used to extract our sample is administered every other year. The following question determined whether or not someone was considered a PWE: “Have you ever been told by a doctor or other health professional that you have a seizure disorder or epilepsy?” [16]. We extend the past research by using a hypothesis-driven approach, multi-year data, and predictive multivariable modeling.

### 2.4. Covariates

The independent variables included health variables, CVD (comprising a positive history of myocardial infarction and stroke), obesity (yes vs. no), chronic kidney disease (CKD) (yes vs. no), and hypertension (yes vs. no), and the following social determinants of healthcare access and utilization: poverty status (<200% Federal Poverty Level), education (no high school, high school, some college, college, and graduate school), race/ethnicity (non-Hispanic white, black, Hispanic, and other), age (years), smoking status (current smoker, former smoker, and non-smoker) alcohol use (never used, former used, and current use), and sex/gender (female vs. male). Unweighted descriptive statistics for respondents who have epilepsy were computed and are reported in Table 1.

The multivariable analyses were weighted using the NHIS-provided weights. We calculated the HR with a 95% confidence interval (CI) for all-cause mortality using Cox proportional hazard regression models. Because our mortality outcome was binary, we used unadjusted and adjusted complex sample Cox regression models for the analysis to predict mortality. Categorical variables were expressed as percentage values and analyzed using chi-square testing. For each participant, the person-time was calculated as the time from the baseline survey participation interview date until the date of death or end of follow-up (31 December 2019), whichever came first. Separate models for sleep deprivation versus all-cause mortality were run by epilepsy status. Additionally, the potential effect of various individual-level differences was analyzed after adjusting for sociodemographic and health factors, estimating variances using the Taylor series linearization method. Individuals surviving beyond the follow-up period were treated as right-censored observations. Additionally, survival curves were generated using the Kaplan–Meier product-limit method to estimate the percent survival of the subject at each point in time. We used Stata 16 for all data management and statistical modeling.

## 3. Results

During follow-up (2.2 million person-years), we identified a total of 6070 mortality cases. Out of 245 participants with seizure disorder, 110 people died (46.5% of the people with seizure disorder vs. 37.2% of the people without seizure disorder, *p* < 0.01). Table 1 elaborates upon the data for the distribution of demographic characteristics of the participants by healthcare provider-diagnosed history of epilepsy using bivariate analysis. The prevalence of seizure disorder in the US adult population in the age group 65 years and older was 1.8%, and the average age of participants with epilepsy was 73.3 years versus 74.6 years in individuals without epilepsy. There was a statistically significant (*p* < 0.05) association between epilepsy and cardiovascular disease, chronic kidney disease (CKD), obesity, age, race/ethnicity, education, poverty–income ratio (PIR), and smoking status. There was also a statistically significant association between epilepsy status (yes 46.5% vs. no 37.2%) and all-cause mortality.

**Figure 1:**
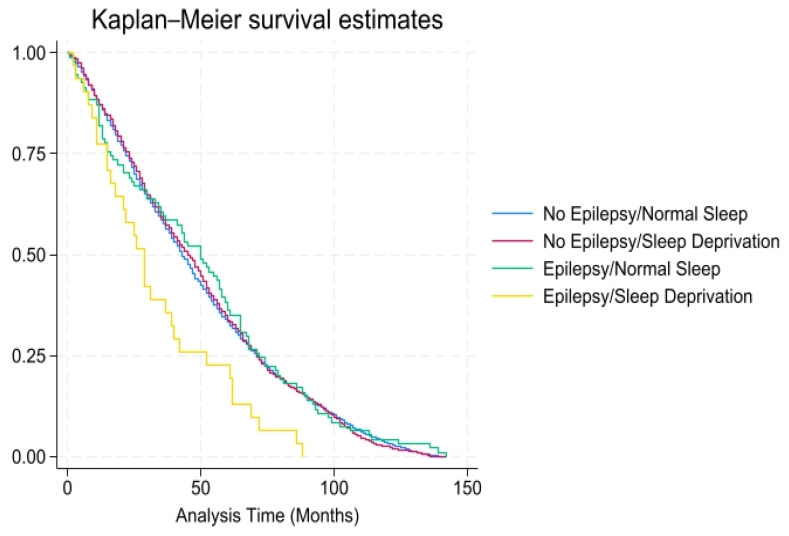
All-cause mortality among individuals with and without epilepsy/sleep deprivation (*x*-axis: time in months; *y*-axis: overall survival probability).

According to Table 2, after 4.8 mean years of follow-up, a higher proportion (46.5% vs. 37.2%, *p* < 0.01) of PWE experienced all-cause mortality than individuals without epilepsy. For all-cause mortality, the unadjusted hazard ratio (HR) for the PWE with sleep deprivation was 1.92 (95% confidence interval [CI 1.09–3.36, *p* < 0.05). The adjusted HR was elevated, 1.94 (CI 1.19–3.15, *p* < 0.01), among those with sleep deprivation but close to 1.0 (1.00 CI 0.78–1.30, *p* = 0.98) among PWE without sleep deprivation, after adjusting for medical (cardiovascular disease, CKD, diabetes, hypertension, obesity, alcohol use, and smoking status) and demographic (ethnicity, education, poverty–income ratio, age, and gender) risk factors. As shown in Figure 1, there was a higher probability of all-cause mortality over time (mean = 10.7 years) among individuals with epilepsy and sleep deprivation.

After 4.8 mean years of follow-up, a higher proportion (46.5% vs. 37.2%, *p* < 0.01) of PWE experienced all-cause mortality than individuals without epilepsy. For all-cause mortality, the unadjusted hazard ratio (HR) for the PWE with sleep deprivation was 1.94 (95% confidence interval [CI], 1.09–3.36, *p* < 0.05). The adjusted HR was elevated, 1.94 (CI 1.19–3.15, *p* < 0.01), among PWE with sleep deprivation but close to 1.0 (1.00 CI 0.78–1.30, *p* = 0.98) among PWE without sleep deprivation, after adjusting for medical (CVD, CKD, diabetes, hypertension, obesity, alcohol use, and smoking status) and demographic (ethnicity, education, poverty-income-ratio, age, and gender) risk factors.

## 4. Discussion

In this large nationally representative study, our primary finding was that people who had both epilepsy and sleep deprivation were more likely to experience mortality than those with each condition individually, especially in the elderly population. Epilepsy and sleep deprivation may have a synergistic effect in leading to overall mortality in this population. We found that there was nearly double the risk of mortality among PWE compared to people without epilepsy among sleep-deprived patients. From previous research, Subota et al. (2023) found that the additive and cumulative effects of aging may also lead to increased mortality compared to one factor alone, among older adults with adult-onset epilepsy, there was an association between sleep disturbances and dementia—with dementia potentially acting as the final common pathway between the connection of epilepsy, sleep, and mortality [29]. More specifically, the elderly population has unique sleep-related changes, such as increased sleep latency, decreased sleep efficiency, and decreased total sleep time, that may further increase the risk of mortality in the presence of conditions such as epilepsy [30]. Some of these changes can result from the pathophysiology of disruptions in circadian rhythms, cortisol excess, and chronic stress in this population. In another review article, Piccenna [31] mentioned how AEDs may have varied effects on the aging vulnerable population, potentially leading to increased risk of death.

Another major finding was that there is a connection between sleep duration and PWE. There was a lower prevalence of healthy sleep duration among PWE (68.6% vs. 74.0%) than those without epilepsy. Our findings are supported by previous research that establishes a lower proportion of healthy sleep among the PWE population than the general population [32]. From a previous nationally representative study, researchers found that individuals with sleep disturbances are 3.7 times more likely to have epilepsy than the general population [33]. Additionally, using the Pittsburgh Sleep Quality Index (PSQI) as a measure, researchers found significantly poorer sleep quality and reduced sleep efficiency in juvenile myoclonic epilepsy (JME) patients compared to a control group [34]. Sleep disturbances have been shown to increase overall mortality in numerous studies.

Also, prescribing practices for epilepsy traditionally have not taken into consideration comorbidities or AED side effects. More specifically, clinicians should take into consideration adults with epilepsy and the indirect impact on sleep due to AED side effects or the co-occurrence of mood disorders. PWE who have co-occurring uncontrolled mood disorders, such as anxiety and depression, can experience increased risk of sleep deprivation. Multiple researchers have found that certain medications with psychotropic properties should be prescribed to older PWE [35,36,37,38]. In addition, clinicians should consider co-occurring mood disorders and prescribe AEDs that have mood-stabilizing properties (lamotrigine and oxcarbazepine) rather than negative psychotropic properties such as levetiracetam and topiramate [39]. However, even newer AEDs such as lamotrigine and carbamazepine have been directly shown to cause sleep disturbances [40]. In a major nationally representative study, depression was found to have a mediating effect in the relationship between sleep deprivation and epilepsy [33]. Depression and anxiety are also causal factors for inadequate sleep and/or poor sleep quality. Inadequate and poor-quality sleep, insomnia, and sleep-disordered breathing can also lead to mood disorders. There is ample evidence that sleep issues can exacerbate existing depression and anxiety—decreasing quality of life. Cognitive behavioral therapy may be a complementary approach to individuals that have contraindications for medication to address both mood disorders and sleep among PWE [41].

Our study also found that more adults with epilepsy reported a longer sleep duration (>8 h per day) than adults without epilepsy. Previous researchers found that more “long-duration sleep” can be associated with increased seizure risk [34], which further supports our study finding. On the contrary, epileptiform discharges and antiepileptic medications may in turn detrimentally impact sleep. While some PWE might sleep longer overall, their sleep quality can be disrupted by seizures occurring during the night, leading to fragmented sleep and daytime fatigue [35]. The PSQI study depicted that almost one-third of epileptic patients suffer from daytime sleepiness. In addition, researchers have also found that some PWE may have over-reported their sleep duration [16]. However, this may be an indication that PWE have excessive daytime sleepiness, especially with refractory epilepsy. Alternatively, excessive daytime sleepiness may be an indication of obstructive sleep apnea (OSA), which often co-occurs with epilepsy. Additionally, multiple Antiepileptic Drugs (AEDs) are associated with weight gain, which could potentially worsen or increase the risk of OSA and increase the long-term potential for outcomes such as mortality [36,37].

### 4.1. Limitations

There are limitations to this work to be kept in mind when interpreting the results. The main limitation of this study is that we could not establish sleep quality in the various specific types of epilepsy and differentiate between adult-onset epilepsy versus epilepsy. Causality cannot be established due to the observational nature of this study. However, we conducted longitudinal research, making this error less likely. Also, there is a potential for recall bias due to the collection of self-reported data. In addition, there is a potential for variations due to the lack of objective measures. Furthermore, social desirability bias may result in inaccurate data, due to respondents answering questions based on what will be viewed favorably by the researcher. Also, the sleep loss frequency information was not included, making this an important area to consider. Side effects of medication could have caused seizures and were not considered either. Also, measurement bias in self-reported sleep data is a limitation due to the subjectivity of the responses. There was also a lack of information on sleep disorders such as OSA and insomnia. There was also a potential reverse causality, as older adults with epilepsy may experience higher mortality due to neurodegenerative processes. Another limitation is that the results were based on data from this specific age group and cannot be generalized to other age groups. Finally, there is no potential way to discern if the sleep disturbances developed as a result of epilepsy or vice versa due to the inability to establish temporality.

### 4.2. Recommendations

Multiple recommendations can come about as a result of this study. First of all, clinicians should identify and address modifiable risk factors associated with poor sleep duration among PWE, which is of tantamount importance [9]. Also, healthcare providers should assess the quality of life of individuals to screen for sleep deprivation and the effects of insomnia. Next, clinicians should include the usage of objectively measured variables such as findings from a sleep study, EEG, behavioral assessments, and related comorbidities [42,43]. Also, primary care physicians should increase PWE patient education by providing recommended levels of amount of sleep for optimal health and well-being. Clinicians also should focus on related conditions such as OSA and consider treatment with Continuous Positive Airway Pressure (CPAP) in addition to treatment with AEDs. Among the aging population, clinicians should consider physiological changes and polypharmacy when prescribing AEDs [44]. These distinct physiological changes should be considered in clinical trials for AEDs as well. Clinical trials should include older adults—a population that is typically excluded due to poor cognition and other comorbid conditions. Another recommendation is to have patients maintain a sleep diary, as poor seizure control may result in poor sleep quality. Major public health organizations should actively engage and increase awareness about improving sleep hygiene and epilepsy individually; however, more needs to be done to address the connection [45,46]. Medical practitioners should be aware of and have more training on the critical role that sleep deprivation plays in exacerbating the occurrence of seizures. Special recognition of a personal and family psychiatric history should be considered at the time of evaluation for prescribing AEDs.

Patients should be counseled on proper sleep hygiene practices, such as a consistent sleep schedule and avoiding stimulants at night [47]. Promoting, through patient education, physical activity and improved sleep quality is necessary among adults with epilepsy. Also, future research should address how sleep deprivation affects people with different types of seizures in various ways [48,49]. Further clarification of the measure of short sleep duration among individuals with chronic diseases is important to determine [50]. Some objective sleep measures have been easily obtained using fitness trackers or wearable devices (internet of things (IoT)), providing relatively consistent and accurate information about the amount of sleep. Finally, future studies should assess the role of OSA in sleep deprivation–mortality linkage.

## 5. Conclusions

Epilepsy continues to be a complex multifaceted disorder that is on the rise in older adults due to an increase in the aging population. Adequate sleep is essential in maintaining neuronal and cellular health. Sleep deprivation may have a serious and detrimental effect on PWE. Furthermore, AEDs may have side effects, such as an increase in weight, OSA, or diabetes, that could further worsen the effects of sleep deprivation among PWE. As practitioners continue to explore the etiology and comorbidities of epilepsy, the consideration of sleep disturbances must play a central role in improving patient quality of life and survival. More screening through the usage of EEGs and sleep studies can improve the prognostic outlook and quality of life.

## Figures and Tables

**Table 1:** Characteristics of study participants stratified by epilepsy diagnosis (PWE).

Characteristics	Total Population (n = 17,319)	PWE (+) (n = 245)	PWE (−) (n = 17,074)
**Sleep Duration**			
Short Duration (<6 h)	7.6 (7.1–8.1)	7.5 (4.6–12.0)	7.6 (7.1–8.1)
Normal Duration (6 to 8 h)	73.9 (73.1–74.8)	68.6 (62.4–74.8)	74.0 (73.2–74.8)
Long Duration (>8 h)	18.5 (17.8–19.2)	23.9 (18.2–29.6)	18.4 (17.7–19.1)
**Diabetes (yes vs. no)**	22.5 (21.8–23.2)	28.1 (22.0–34.3)	22.4 (21.7–23.1)
**Cardiovascular Disease** ** **(yes vs. no)**	34.4 (33.6–35.2)	50.7 (43.2–58.1)	34.2 (33.3–35.0)
**Smoking Status** **			
Never Smoked	50.9 (50.0–51.8)	37.6 (30.8–44.4)	51.1 (50.1–52.0)
Formerly Smoked	39.4 (38.5–40.3)	48.0 (40.5–55.6)	39.3 (38.4–40.2)
Current Smoker	9.7 (9.1–10.2)	14.4 (9.4–19.3)	9.6 (9.1–10.2)
**Alcohol Use** **			
Never Used	27.8 (26.9–28.8)	31.4 (24.2–38.6)	27.8 (26.8–28.8)
Former Use	26.6 (25.8–27.5)	36.2 (29.0–43.3)	26.5 (25.6–27.4)
Current Use	45.5 (44.5–46.5)	32.5 (25.1–39.8)	45.7 (44.6–46.7)
**Chronic Kidney Disease (CKD)** ** **(yes vs. no)**	4.4 (4.1–4.7)	9.3 (6.6–13.1)	4.3 (4.0–4.7)
**Obesity** * **(yes vs. no)**	25.3 (24.6–26.0)	31.8 (25.4–38.2)	25.2 (24.5–25.9)
**Hypertension (yes vs. no)**	37.3 (36.4–38.2)	36.6 (29.6–43.6)	37.3 (36.4–38.2)
**Age (SE)** **	74.6 (0.07)	73.3 (0.48)	74.6 (0.08)
**Gender (Male %)**	39.2 (38.4–40.2)	44.1 (37.5–50.6)	39.2 (38.4–39.9)
**Poverty–Income Ratio** (<2) *	11.7 (11.0–12.60	17.6 (12.1–23.2)	11.7 (10.9–12.5)
**Ethnicity** **			
Non-Hispanic White	80.7 (79.9–81.6)	76.3 (71.2–81.5)	80.8 (80.0–81.6)
Non-Hispanic Black	9.4 (8.7–10.1)	15.8 (11.7–20.0)	9.3 (8.6–10.0)
Hispanic	6.9 (6.5–7.4)	5.3 (3.2–8.7)	6.9 (6.5–7.4)
Asian	3.0 (2.7–3.3)	2.5 (1.2–5.4)	3.0 (2.7–3.3)
**Education Level** **			
Some High School	22.4 (21.5–23.3)	32.6 (26.0–39.3)	22.3 (21.4–23.2)
High School Graduate	31.9 (31.1–32.8)	29.4 (23.0–35.8)	32.0 (31.1–32.8)
Some College	23.9 (23.2–24.7)	16.6 (11.3–22.0)	24.0 (23.3–24.8)
College Graduate and Beyond	21.7 (20.8–22.6)	21.3 (14.8–27.8)	21.7 (20.8–22.6)
**All deaths** (N, %) **	37.3% (6070)	46.5% (110)	37.2% (5960)

Note. * *p* < 0.05, ** *p* < 0.01. Numbers with 95%CI indicate 95% confidence intervals for proportions.

**Table 2:** Risk of all-cause mortality among older adults with epilepsy based on sleep deprivation using adjusted Cox proportional hazard model.

	Total Population HR (95%CI)	No Epilepsy Sleep Deprivation HR (95%CI)	Epilepsy No Sleep Deprivation HR (95%CI)	Both Epilepsy and Sleep Deprivation HR (95%CI)
**PWE/Sleep**	1.14 (0.89–1.45)	0.99 (0.99–1.09)	1.00 (0.78–1.30)	1.94 (1.19–3.15) **
**Diabetes**	0.97 (0.88–1.06)	0.96 (0.88–1.050	0.97 (0.87–1.08)	0.95 (0.82–1.11)
**Cardiovascular Disease**	1.18 (1.09–1.27) **	1.17 (1.09–1.27) **	1.13 (1.04–1.23) **	1.32 (1.11–1.56) **
**Smoking Status**				
Never Smoked	Ref	Ref	Ref	Ref
Formerly Smoked	1.07 (0.99–1.17)	1.07 (0.98–1.16)	1.06 (0.96–1.17)	1.11 (0.93–1.32)
Current Smoker	1.18 (1.05–1.33) **	1.18 (1.05–1.32) **	1.11 (0.97–1.26)	1.44 (1.12–1.84) **
**Alcohol Use**				
Never Used (Ref)	Ref	Ref	Ref	Ref
Former Use	1.10 (1.01–1.20) *	1.10 (1.01–1.21) *	1.12 (1.00–1.25)	1.03 (0.85–1.26)
Current Use	0.96 (0.88–1.05)	0.96 (0.88–1.25)	0.95 (0.86–1.06)	1.01 (0.82–1.23)
**Chronic Kidney Disease**	1.44 (1.26–1.64) **	1.46 (1.28–1.66) **	1.51 (1.28–1.79) **	1.32 (1.04–1.68) *
**Obesity**	1.04 (0.95–1.13)	1.04 (0.95–1.13)	1.05 (0.95–1.17)	1.01 (0.85–1.18)
**Hypertension**	1.01 (0.93–1.10)	1.00 (0.93–1.09)	1.00 (0.91–1.09)	1.06 (0.90–1.25)
**Age**	1.02 (1.02–1.03) **	1.02 (1.02–1.03) **	1.02 (1.01–1.03) **	1.02 (1.01–1.04) **
**Gender (Ref Male)**	0.93 (0.86–1.01)	0.93 (0.86–1.01)	0.92 (0.84–1.01)	0.99 (0.85–1.16)
**Poverty–Income Ratio** (Ref: PIR < 2)	1.12 (1.02–1.24) *	1.12 (1.01–1.25) *	1.05 (0.93–1.19)	1.30 (1.08–1.56) **
**Ethnicity**				
Non-Hispanic White	Ref	Ref	Ref	Ref
Non-Hispanic Black	0.99 (0.90–1.09)	0.98 (0.89–1.09)	1.06 (0.94–1.19)	0.95 (0.70–1.04)
Hispanic	0.96 (0.82–1.12)	0.95 (0.82–1.10)	0.91 (0.78–1.08)	1.03 (0.76–1.40)
Asian	0.95 (0.80–1.12)	0.95 (0.80–1.12)	0.98 (0.81–1.19)	0.87 (0.65–1.17)
**Education Level**				
College Grad or Higher	Ref	Ref	Ref	Ref
Some High School	0.98 (0.88–1.10)	0.99 (0.89–1.11)	1.05 (0.92–1.20)	0.86 (0.68–1.08)
High School Graduate	0.97 (0.87–1.08)	0.97 (0.87–1.08)	0.97 (0.86–1.10)	1.00 (0.77–1.27)
Some College	0.91 (0.81–1.02)	0.91 (0.82–1.02)	0.89 (0.79–1.01)	0.97 (0.74–1.28)

Note. * *p* < 0.05; ** *p* < 0.01. HR (95%CI) indicates hazard ratios with 95% confidence intervals for the outcome (i.e., mortality). Ref indicates the reference group among each variable for comparison with other groups.

## Data Availability

The data presented in this study are openly available in [NHANES] at [https://www.cdc.gov/nchs/nhanes/?CDC_AAref_Val=https://www.cdc.gov/nchs/nhanes/index.htm], accessed on 31 December 2024.
